# Case Report: Antiangiogenic Therapy Plus Immune Checkpoint Inhibitors Combined With Intratumoral Cryoablation for Hepatocellular Carcinoma

**DOI:** 10.3389/fimmu.2021.740790

**Published:** 2021-10-18

**Authors:** Xin Li, Jiahua Xu, Xiaoqiang Gu, Ling Chen, Qing Wu, Hongwei Li, Haoran Bai, Jinzu Yang, Jianxin Qian

**Affiliations:** ^1^ Department of Oncology, Longhua Hospital Affiliated to Shanghai University of Traditional Chinese Medicine (TCM), Shanghai, China; ^2^ Department of Oncology, Yueyang Hospital of Integrated Traditional Chinese and Western Medicine Affiliated to Shanghai University of Traditional Chinese Medicine (TCM), Shanghai, China

**Keywords:** hepatocellular carcinoma, cryoablation, immune checkpoint inhibitors, antiangiogenic therapy, case report

## Abstract

**Background:**

Hepatocellular carcinoma (HCC) is a common gastrointestinal malignancy with high incidence and poor prognosis. Common treatment methods include surgery, transcatheter arterial chemoembolization (TACE), ablation, and targeted therapy. In recent years, combination treatment with antiangiogenic therapy and immune checkpoint inhibitors has made great progress in the treatment of advanced HCC. Here, we report the case of a patient with HCC who achieved a durable benefit from anti-vascular therapy and immune checkpoint inhibitors combined with intratumoral cryoablation.

**Main Body:**

A 38-year-old male patient initially presented with severe abdominal pain that was identified as an HCC rupture and hemorrhage by computed tomography (CT). The patient underwent emergency surgery and postoperative pathology confirmed HCC. The patient received prophylactic TACE after surgery. Unfortunately, three months after surgery, the patient developed multiple liver metastases. Subsequently, he received systemic anti-vascular therapy and immune checkpoint inhibitors combined with intratumoral cryoablation. After treatment, the patient achieved extensive tumor necrosis and the disease was effectively controlled.

**Conclusions:**

Anti-angiogenic therapy and immune checkpoint inhibitors combined with cryoablation can induce a powerful and effective systemic anti-tumor immune response, which is worthy of further research.

## Introduction

Liver cancer is a common gastrointestinal malignancy that is associated with a poor prognosis. Currently, among all cancers, liver cancer ranks 6th for incidence and 3^rd^ for mortality globally (1). The two main liver cancer subtypes are hepatocellular carcinoma (HCC) and intrahepatic cholangiocarcinoma, of which HCC accounts for more than 90% of primary tumors of the liver. At present, treatment methods for HCC include surgical therapy, transcatheter arterial chemoembolization (TACE), ablation therapy, molecular targeted therapy and immunotherapy (2). However, a majority of patients with HCC are detected at an advanced stage that precludes surgical treatment and for patients able to undergo surgical intervention the postoperative recurrence rate is high. This limits the application of surgical therapy and contributes to the poor clinical prognosis for HCC.

For patients with advanced HCC with extrahepatic spread, treatment is usually based around the molecular targeted therapies sorafenib and lenvatinib. In recent years, the development of immunotherapies such as immune checkpoint inhibitors have also led to great progress in the treatment of HCC (3). In particular, based on the results of the IMbrave150 trial, combination treatment with atezolizumab (an immune checkpoint inhibitor) and bevacizumab (an anti-angiogenic agent) has been approved as a first-line treatment for advanced HCC and is recommended by the National Comprehensive Cancer Network treatment guidelines in this setting (4). However, although immune checkpoint inhibitors have achieved good outcomes in HCC, the application of immunotherapy in HCC still faces many challenges such as the relatively low response rate characteristic of this class of therapy and a lack of tumor biomarkers to identify treatment-sensitive patients.

Image-guided tumor ablation (radiofrequency, microwave or cryoablation) has been applied to numerous cancers, including renal cell carcinoma, prostate cancer, lung cancer, and liver cancer with promising short-term results. However, cryoablation has several comparative advantages over radiofrequency ablation and other thermal-based ablation approaches, including the ability to produce a larger and more precise ablation area, a more clearly identifiable therapeutic area, and the ability to stimulate immune regulation to produce ectopic tumor suppressive effects (5, 6).

Here, we report the case of a patient with HCC diagnosed as the result of tumor rupture and hemorrhage who experienced a sustained survival benefit following treatment with antiangiogenic and immunotherapy combined with intratumoral cryoablation after post-surgical tumor recurrence. To our knowledge, this treatment strategy has not been previously reported.

## Case Description

**Figure 1:**
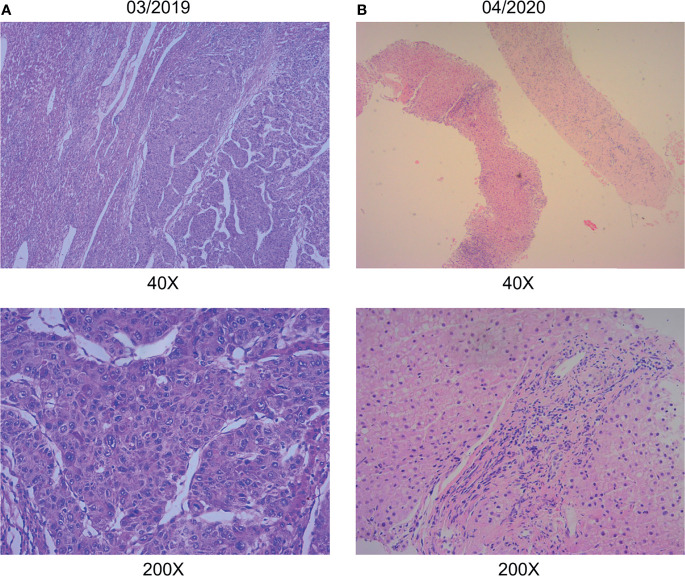
Pathological data. **(A)** Hepatic surgery specimens showed histological characteristic of hepatocellular carcinoma. **(B)** Liver puncture specimens showed no histological characteristic of malignancy. (hematoxylin-eosin stain; original magnification 40×and 200×).

**Figure 2:**
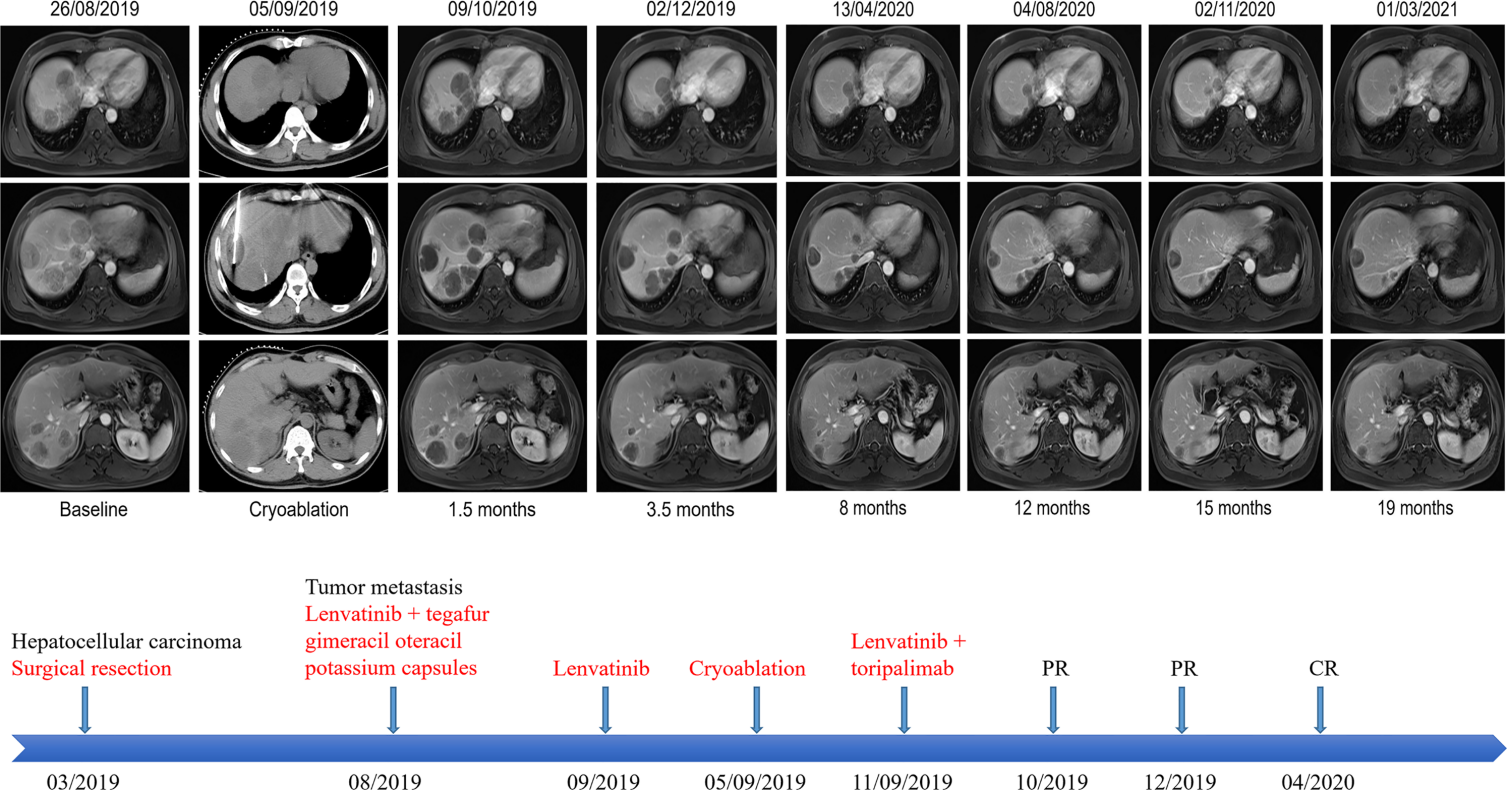
Representative upper abdominal imaging showing changes in the liver tumor throughout the treatment period and the patient’s course of treatment. After intratumoral cryoablation combined with antiangiogenic therapy plus immune checkpoint inhibitors, the patient’s liver tumor was necrotic, with no obvious tumor activity, and some tumor lesions disappeared and shrank, which were evaluated as a complete response according to mRECIST.

A 38-year-old Chinese male patient presented to the hospital in March 2019 with severe abdominal pain. Computed tomography (CT) imaging indicated that the patient had a ruptured liver tumor in the lower right lobe of the liver and was experiencing bleeding. The patient had a history of chronic viral hepatitis B (HBV) with no previous antiviral therapy. His mother and brother also had a history of chronic HBV. The patient had no family history of cancer. The patient underwent an emergency liver tumor resection on March 2019. A postoperative pathological diagnosis revealed HCC with tumor thrombus in the vessel (**Figure 1A**). Hepatic arteriography was performed one month after surgery, and no tumor staining was observed in the liver. In addition, Positron Emission Tomography-CT (PET-CT) indicated no systemic metastasis after resection of the tumor in the lower right lobe of the liver. A time course of the entire case is shown in **Figure 2**.

## Diagnostic Assessment

On August 26, 2019, the patient underwent routine follow-up with an enhanced magnetic resonance imaging (MRI) of the upper abdomen, which revealed multiple metastatic tumors in the liver, the larger ones were around 6.3 x 4.3 x 4.7 cm, with hepatic hilar lymph node metastasis, suggesting that the patient’s Barcelona Clinic Liver Cancer (BCLC) stage was stage C (**Figure 2**). At this time, the patient’s alpha fetoprotein (AFP) was 6050 ng/mL. Subsequently, the patient underwent a liver tumor puncture biopsy and next generation sequencing (NGS) was performed on the puncture sample in a College of American Pathologists accredited laboratory (GeneCast Biotechnology Co, Beijing). The test results indicated that the patient had negative programmed cell death ligand 1 (PD-L1) expression, a microsatellite status of MS-Stable, and the tumor mutation burden (TMB) was 3 Muts/Mb. The gene mutation results are summarized in **Table 1**. The patient subsequently initiated treatment with lenvatinib (12 mg/day) in combination with tegafur gimeracil oteracil potassium capsules (60mg twice daily), but he developed severe nausea and vomiting.

**Table 1:** Summary of genetic test results.

Pre-cryoablation			Post-cryoablation		
GENES	Variations	Abundance	GENES	Variations	Abundance
KLHL6	p.T328M	1.63%	ABCC1	p.C1479Y	0.57%
MLL2	p.L4575I	11.48%	ABCC4	p.A971T	0.55%
MLL2	p.T4110I	32.73%	AURKB	p.A329T	2.50%
MUTYH	Q253*		AXIN1	p.Q184Rfs*58	0.90%
NTRK	p.N218K	1.17%	CDK9	p.R188H	0.81%
RICTOR	amplification		CREBBP	p.R1347W	0.50%
TP53	p.R249S	5.56%	DOT1L	p.A1254T	1.13%
TSC2	loss		EGFR	p.A767V	0.54%
MSI	MS-stable		FH	p.V73M	0.53%
TMB	3 Muts/Mb		FLT3	p.G891D	1.53%
			FOXA1	p.E456G	0.54%
			GLI2	p.A43V	2.62%
			GLI2	p.R1470W	0.68%
			HGF	p.G396D	1.13%
			IRS2	p.A618V	1.61%
			KEAP1	p.Q20*	0.96%
			KMT2B	p.C1249Y	0.67%
			LZTR1	p.A512T	1.11%
			MUTYH	P.Q264*	
			NKX2-1	p.G134D	0.79%
			NOTCH4	p.P237S	0.57%
			NSD1	p.A955V	0.52%
			PARP4	p.T1170I	6.11%
			PRF1	p.G149D	0.86%
			RBM10	p.A717V	0.70%
			SLC16A7	p.L241P	3.82%
			SMARCA1	p.G889D	0.58%
			SOX9	p.A209V	0.51%
			TERT	p.A745V	0.55%
			MSI	MS-stable	
			TMB	18.67 Muts/Mb	

* stands for codon variation variation.

**Figure 3:**
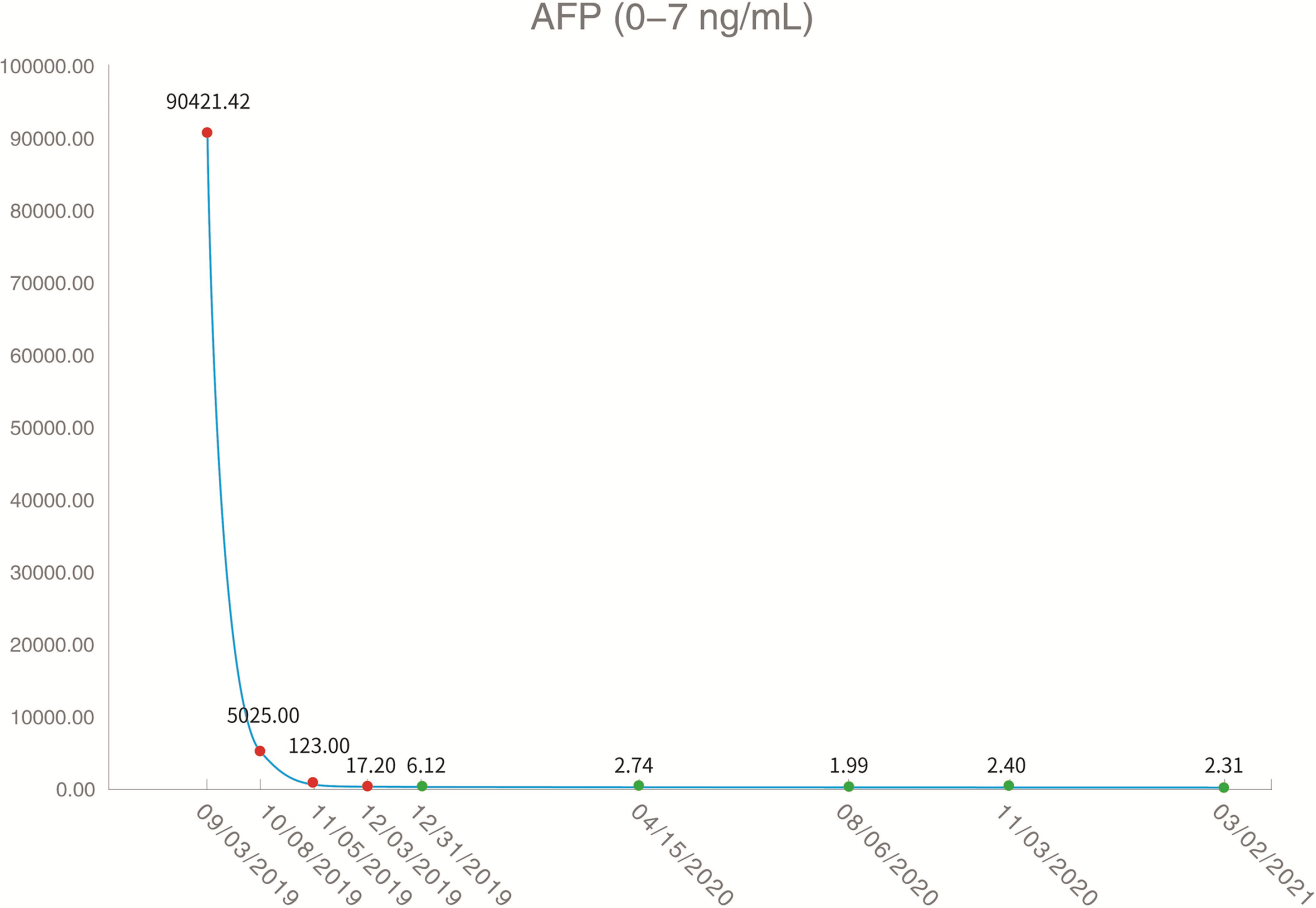
Serum alpha fetoprotein levels throughout the treatment period (red dots indicate increased and green dots indicate normal levels).

The patient came to our hospital in early September 2019, and at this time, the patient’s alpha fetoprotein (AFP) level was 90421.42 ng/mL (**Figure 3**). Considering that the nausea and vomiting may be mainly caused by the tegafur gimeracil oteracil potassium capsule, we suggested that the patient stop taking tegafur gimeracil oteracil potassium capsules, but continue to be treated with low dose lenvatinib (8 mg/day). On September 5, we performed CT-guided intratumoral cryoablation of one large metastatic tumor in the patient’s liver (other lesions and lymph nodes did not receive cryoablation). A postoperative CT scan revealed complete ablation of the tumor. On the fourth day after cryoablation, we performed a CT-guided puncture biopsy on the metastatic liver tumor that had not received cryoablation, and performed NGS testing on the tissue sample and peripheral blood plasma at GeneCast Biotechnology Co. The NGS sequencing results showed negative PD-L1 expression with MS-Stable microsatellite status and TMB of 18.67 Muts/Mb. The gene mutation results are summarized in **Table 1**. On September 11, the patient initiated treatment with lenvatinib (8 mg/day) in combination with the immune checkpoint inhibitor toripalimab (240 mg IV infusion every 3 weeks), a humanized monoclonal antibody targeting the programmed cell death 1 (PD-1) receptor. On October 8, the AFP of the patient dropped to 5025.00 ng/mL. An MRI of the upper abdomen indicated that after the comprehensive treatment of the liver tumors, there were multiple masses in the liver. Considering the partial survival of the tumor, the tumor necrosis was more obvious than that in the previous image.

Encouraged by these exceptional results, a decision was made to continue systemic treatment with combined lenvatinib (8 mg orally daily) and toripalimab (240 mg IV infusion every 3 weeks). In early December 2019, the patient’s AFP level had dropped to 17.20 ng/mL. MRI of the upper abdomen showed multiple abnormal signals and partial abnormal enhancement in the liver. Considering most of the tumor necrosis and partial survival, the tumor volume was reduced compared with the previous images taken on 2019-10-09.

Thereafter, the patient continued lenvatinib and toripalimab. An upper abdominal MRI acquired in April 2020 revealed necrosis of the tumor and continued reductions in tumor volume. According to the modified response evaluation criteria in solid tumors (mRECIST), the patient had achieved a complete response (CR). In order to evaluate the extent of residual active tumor tissue in the liver of the patient, we conducted a puncture biopsy on the suspected active tumor under the guidance of B-ultrasound. Pathological assessment of the puncture biopsy samples revealed local hepatocyte hyperplasia with edema and degeneration, and fibrous tissue hyperplasia in some areas. No clear evidence of malignant tumor was found (**Figure 1B**). The patient has continued this combined treatment regimen until now (last treatment date July 11, 2021), and their AFP level in regular examinations has been consistently below the normal value (0-7 ng/mL). A PET-CT examination in August 2021 showed multiple nodules in the liver, decreased FDG metabolism indicating no active tumor in the liver, and no tumor metastasis in the whole body (**Supplementary Figure 1**).

During the whole treatment period, the patient had no obvious discomfort, no adverse events related to antiangiogenic therapy or immunotherapy, and their HBV replication level was well controlled.

## Discussion

Here, we present the case a patient with metastatic HCC who had disease recurrence following surgery and then received cryoablation followed by combined lenvatinib and toripalimab. The patient achieved a complete response after 7 months of treatment which has been sustained until the submission of this manuscript, giving a progression free survival of 24 months at the last calculation.

The tumor microenvironment is a highly heterogeneous microecosystem composed of tumor cells and their surrounding environment, which is characterized by tissue ischemia, hypoxia and low pH, and evolves with the development of tumor (7, 8). Normalizing the tumor microenvironment to enhance anticancer therapy is currently a research topic of great interest. In particular, a large number of studies have demonstrated that antiangiogenic therapy combined with immunotherapy can normalize tumor vasculature and thus alleviate ischemia and hypoxia in the tumor microenvironment, increase the delivery of drugs and anti-tumor immune cells, and transform the tumor immunosuppressive microenvironment into an immune-supportive microenvironment (9–11). Based on the above mechanisms, anti-angiogenic therapy combined with immunotherapy (atezolizumab plus bevacizumab) has achieved breakthrough progress in the treatment of HCC, and has since been recommended as a first-line treatment for patients with advanced HCC (12). Lenvatinib is a small molecule targeted antiangiogenic agent that is approved for the first-line treatment of HCC. Toripalimab has also been shown to have excellent and durable antitumor activity against a variety of advanced or recurrent malignancies, including HBV-associated HCC (13, 14). Therefore, we treated this patient with lenvatinib combined with toripalimab with the expectation of a robust and durable therapeutic effect.

Cryoablation is a locoregional treatment for tumor lesions that is often used in patients with HCC. Studies have shown that cryoablation is safe and significantly improved local control of HCCs >2 cm in diameter compared with radiofrequency ablation and microwave ablation (15). More importantly, cryoablation can produce an abscopal effect, which is thought to affect cancer cells outside the primary ablation area. At present, the mechanism of abscopal effect in cryoablation is not clear. Cryoablation of the tumor tissue results in cell destruction by freezing. Contrary to heat-based ablative modalities, cryoablation induces tumor cell death by osmosis and necrosis. It is hypothesized that with necrosis, the intracellular contents of the cancer cells stay intact, and that these contents are mechanically similar to vaccination, where hundreds of unique tumor-derived self-antigens are released into circulation (16, 17). However, the release of tumor-derived self-antigens into the circulation alone may not be sufficient to overcome tumor immune escape. Simultaneous boosting of the immune response through the use of immune checkpoint inhibitors may therefore be the optimal treatment strategy to enable the immune system to recognize these novel circulating self-antigens. This hypothesis is supported by multiple studies showing that cryoablation can upregulate the expression of circulating PD-L1/PD-1 (18), and induce a more potent immune response compared with other local treatments such as radiofrequency ablation (19). Therefore, cryoablation combined with immune checkpoint inhibitors is considered to have a synergistic effect (20). Some preclinical and clinical evidence has shown that cryoablation combined with immunotherapy can produce synergies, generating an abscopal effect to distant lesions (21–24). However, this reaction is idealistic and is rarely observed in clinical practice. In the present case, the patient underwent NGS sequencing after cryoablation, and the results showed that the patient’s TMB was significantly higher than observed following initial surgical resection, and the number of mutated genes was also significantly increased. We speculated that this may be a manifestation of novel cryoablated self-antigens formed by the intracellular contents of the cancer cells entering the circulation system after cryoablation.

There have been preliminary studies of the effect of cryoablation on the activation of the immune system, but the effect of cryoablation on tumor blood vessels is less well described. It has been reported that cryoablation of normal rat skin can stimulate local angiogenesis, but the resulting neovasculature may not be fully functional (25). Some studies have found that the expression of vascular endothelial growth factor (VEGF) is up-regulated in tumor tissue after cryoablation, and angiogenesis in residual tumor is significantly increased. Anti-angiogenic therapy can downregulate the expression of VEGF, inhibit tumor angiogenesis, and therefore improve the inhibitory effect of cryoablation on tumors (26, 27). It is well known that in the tumor vascular system, the vascular structure is disordered, the morphology is abnormal, and the structure of vascular walls is incomplete, leading to leakage of the vascular wall, elevated interstitial fluid pressure, and elevated blood flow resistance (28). We therefore hypothesized that new blood vessels formed after cryoablation will also have these characteristics and therefore antiangiogenic therapy combined with cryoablation may also have a synergistic effect. However, the choice of dosage for antiangiogenic therapy is very important.

High-dose antiangiogenic therapy may over-prune tumor vessels, which may adversely affect the delivery of systemic therapies, while low-doses of antiangiogenic therapy can normalize tumor blood vessels, and increase the delivery of anti-tumor drugs and immune cells (29), and at the same time allow the cryoablated self-antigens to efficiently enter the circulation, thus stimulating strong tumor immunity. In addition, the order of drug administration also plays a very important role. Experiments have shown that sequential immunotherapy or chemotherapy after antiangiogenic therapy generates a vascular normalization window and has a better therapeutic effect (28, 30).

A key limitation of this case study is that the patient underwent an emergency surgical resection of HCC and may not represent the usual population of patients with HCC who undergo surgery. In summary, the patient in this case received intratumoral cryoablation after low-dose antiangiogenic therapy. We hypothesize that the normalized tumor blood vessels facilitated entry of cryoablated self-antigens into the circulation as well as promoting the formation of an immune-supportive tumor microenvironment, which was then followed by the administration of immunotherapy to the patient. The three synergistic effects of antiangiogenic therapy, intratumoral cryoablation, and immunotherapy resulted in a very positive outcome for the patient. However, the mechanism of this synergistic therapy is still unclear, and there are no clear criteria for the selection of drug dosage, sequence of drug administration and time point of cryoablation, which are worthy of further study.

## Patient Perspective

The written informed consent of the patient was obtained for the publication of this case report and any identifying information and images. He was happy to agree to publication of this case report in hopes of furthering medical knowledge in this area.

## Data Availability Statement

The raw data supporting the conclusions of this article will be made available by the authors, without undue reservation.

## Ethics Statement

Ethical review and approval were not required for the study on human participants in accordance with the local legislation and institutional requirements. The patients/participants provided their written informed consent to participate in this study. Written informed consent was obtained from the individual(s) for the publication of any potentially identifiable images or data included in this article.

## Author Contributions

XL, JX, XG, and LC contributed equally to this work and are co-first authors. QW, JY, and JQ designed the study. XL and JX collected the data. XL and LC drafted the manuscript. XG, JX, and LC performed radiological data analysis and provided the imaging figures. HL and HB analyzed the data. All authors contributed to the article and approved the submitted version.

## Funding

This work was partially supported by Genecast Cancer Research Project of Beijing CSCO Clinical Cancer Research Foundation (Y2019Genecast-074) and Natural Science Foundation of Shanghai (21ZR1463600). The funder was not involved in the study design, collection, analysis, interpretation of data, the writing of this article or the decision to submit it for publication.

## Conflict of Interest

The authors declare that the research was conducted in the absence of any commercial or financial relationships that could be construed as a potential conflict of interest.

## Publisher’s Note

All claims expressed in this article are solely those of the authors and do not necessarily represent those of their affiliated organizations, or those of the publisher, the editors and the reviewers. Any product that may be evaluated in this article, or claim that may be made by its manufacturer, is not guaranteed or endorsed by the publisher.

## Supplementary Material

Supplementary Figure 1 PET-CT examination conducted in August 2021 showing multiple nodules in the liver and decreased FDG metabolism, indicating no active tumor in the liver.
