# Supplementary figures and images for: Case Report: Antiangiogenic Therapy Plus Immune Checkpoint Inhibitors Combined With Intratumoral Cryoablation for Hepatocellular Carcinoma
Source: Front Immunol. 2021 Oct 18;12:740790. doi: 10.3389/fimmu.2021.740790 (PMC8559549; doi:10.3389/fimmu.2021.740790)

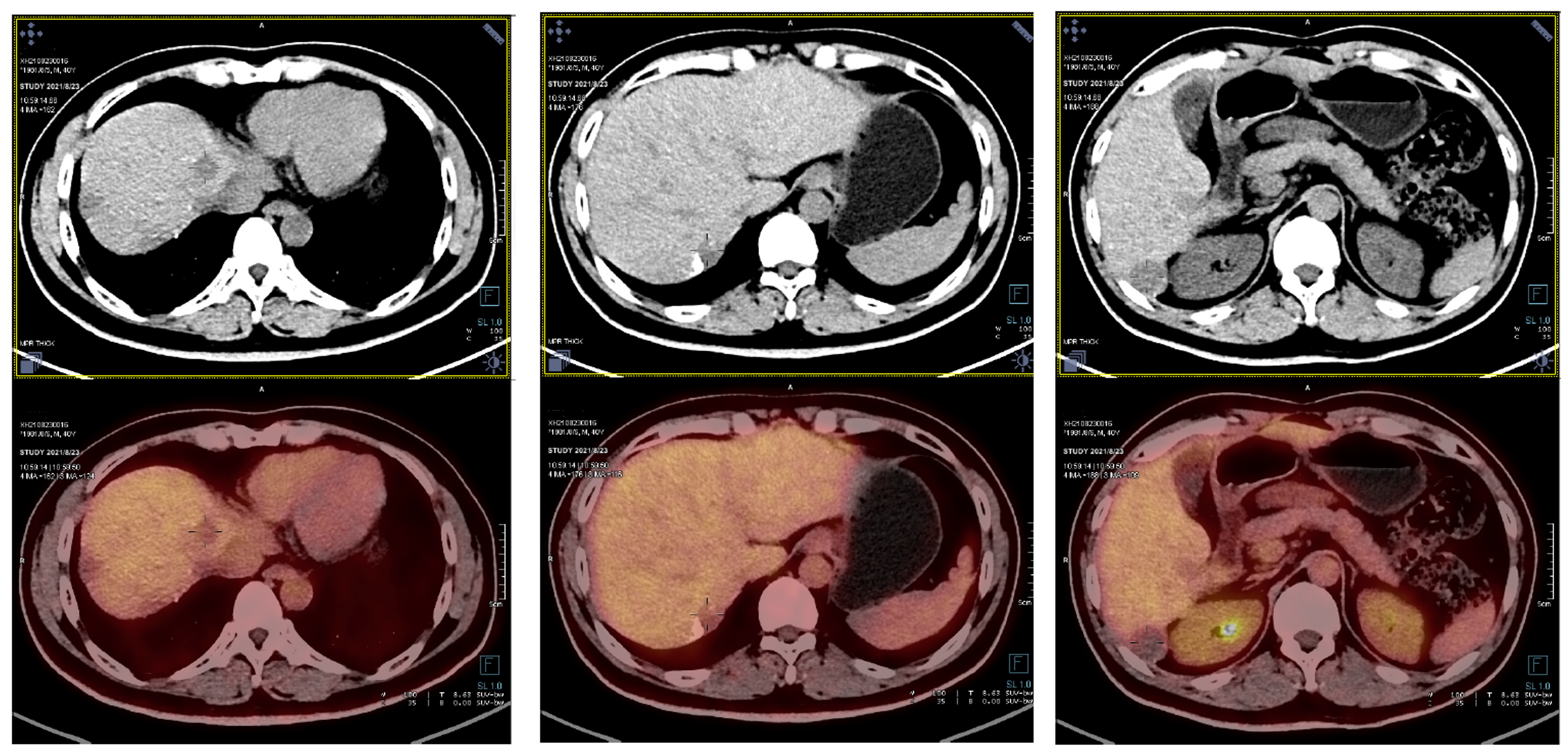

Supplement: Supplementary Figure 1 — PET-CT examination conducted in August 2021 showing multiple nodules in the liver and decreased FDG metabolism, indicating no active tumor in the liver. [file Image_1.tif]
